# Pavlovian-instrumental transfer effects in individuals with binge eating

**DOI:** 10.1186/s40337-023-00824-w

**Published:** 2023-07-06

**Authors:** Wai Sze Chan, Tsun Tak Lai

**Affiliations:** Department of Psychology, The University of Hong Kong, Rm 664, The Jockey Club Tower, Centennial Campus, Pokfulam, Hong Kong SAR

**Keywords:** Binge eating disorder, Bulimia nervosa, Food addiction, Cue reactivity, Food reward, Motivational biases, Reward sensitization

## Abstract

**Background:**

The food addiction model of binge-eating postulates that hyperpalatable food can sensitize the reward processing system and lead to elevated cue-elicited motivational biases towards food, which eventually become habitual and compulsive. However, previous research on food reward conditioning in individuals with binge-eating is scarce. The present study examined the Pavlovian-instrumental transfer (PIT) effects in individuals with recurrent binge-eating. It was hypothesized that hyperpalatable food would elicit specific transfer effects, i.e., biased responding for the signaled food even after satiation on that food, and this effect would be stronger in individuals with binge-eating compared to healthy controls.

**Methods:**

Fifty-one adults with recurrent binge-eating and 50 weight-matched healthy controls (mean age: 23.95 [SD = 5.62]; % female = 76.2%) completed the PIT paradigm with food rewards. Participants also completed measures of hunger, mood, impulsivity, response disinhibition, and working memory. Mixed ANOVAs were conducted to examine transfer effects and if they differed between individuals with binge-eating and those without.

**Results:**

The group by cue interaction effect was not significant, suggesting that the specific transfer effect did not differ between groups. The main effect of cue was significant, indicating that the outcome-specific cue biased instrumental responding towards the signaled hyperpalatable food. However, the biased instrumental responding was attributable to suppressed responding in the presence of the cue predicting no reward, rather than enhanced responding in the presence of the specific food-predicting cues.

**Conclusions:**

The present findings did not support the hypothesis that individuals with binge-eating would be more vulnerable to specific transfer effects elicited by hyperpalatable food, as measured by the PIT paradigm.

## Abstract (plain language summary)

Cues associated with food are known to increase one’s motivation for food consumption. Such a tendency, if not diminished after satiation, may lead to excessive and compulsive food consumption. Hyperpalatable food, which refers to food high in fats, refined carbohydrates, sugar, and sodium, is believed to elevate food-consuming motivation and may lead to binge-eating behavior. Hence, the present study aimed to evaluate the effects of cues on people’s motivation to seek signaled hyperpalatable food in those with or without recurrent binge-eating. Results showed that, although conditioned cues biased the choice of response towards the signaled food, this bias was due to suppressed responding in the presence of cues predicting no reward rather than increased responding in the presence of cues predicting the signaled reward. Notably, we did not find significant differences in this effect between people with binge-eating and those without, suggesting that the effects of cues on food-seeking motivation did not differ in people with recurrent binge-eating.

## Introduction

### Background

Binge-eating refers to the consumption of an excessive amount of food within a short period, accompanied by feeling a loss of control over eating [1]. Binge-eating is a defining feature in individuals with binge eating disorder (BED), which is diagnosed by the presence of recurring binge-eating episodes without any compensatory behaviors once or more per week for at least three months. These binge-eating episodes are associated with at least three of the following: eating more rapidly than normal, eating until uncomfortably full, eating large amounts of food despite not feeling hungry, eating alone due to feeling embarrassed about the amount eaten, and feeling disgusted with oneself, depressed, or guilty after overeating [2]. Binge-eating is also present in bulimia nervosa (BN), which is diagnosed by the overvaluation of weight and body shape and recurring binge-eating episodes in conjunction with compensatory behavior, such as self-induced vomiting, misuse of laxatives, diuretics, or other medications, fasting or excessive exercise, for at least once per week for three months [2]. Eating disorders are associated with elevated mortalities, economic costs, and long-term negative consequences on the health and quality of life of individuals, caregivers, and families [3]. BED, in particular, has the highest prevalence of up to 3.5%, is highly comorbid with other psychiatric disorders, and is associated with numerous medical complications commonly seen not only in eating disorders but also in obesity [4].

The addiction model has been recently adopted to conceptualize binge-eating because of the phenotypic overlap between binge-eating and addiction and their common neurobiological underpinnings [5–7]. For instance, individuals with binge-eating experience food craving, compulsive food consumption despite negative consequences, repeated failed attempts to stop excessive food consumption, and social and functional impairments, which are similar to the symptoms of substance addiction [5, 7]. Altered neurobiological processes related to reward processing, inhibitory control, and habit formation are implicated in both binge-eating and addiction [6]. Foods consumed during a binge episode are often hyperpalatable [8], which share similarities with addictive drugs in that it has unnaturally high rewarding properties and can rapidly activate the reward circuitry in the brain [5]. Although its construct validity and utility in explaining overeating in the general population remain controversial [9], food addiction is considered a plausible model for conceptualizing binge-eating pathology [6, 7]. Nonetheless, it is well-recognized that more research is needed to examine whether mechanistic processes underlying binge-eating share similarities with those underlying addiction [5, 10].

The vulnerability to reward cue sensitization is one of the mechanisms postulated to underlie the pathophysiology of both addiction and binge-eating. In the literature examining substance addiction, the incentive sensitization theory (IST) proposes that repeated exposure to drugs sensitizes the mesolimbic system to drug-associated cues and leads to heightened motivation for drug-seeking behavior that eventually becomes compulsive, i.e., perseverant despite changes in the outcomes, such as increasing negative consequences and diminishing rewarding values [11, 12]. This transition from the initial voluntary, instrumental drug-taking behavior to habitual, compulsive behavior is paralleled by the neurological changes indicating a transition from prefrontal to striatal control and from ventral to dorsal striatal control over instrumental actions [13]. In the food addiction model of binge-eating, hyperpalatable food is postulated to act like addictive drugs that can sensitize the mesolimbic system [5], leading to elevated cue-elicited motivational biases for these foods, and this motivational bias is suggested to become insensitive to outcome devaluation, constituting to the loss of control over eating [6, 7].

Previous studies have found robust evidence for cognitive biases towards food stimuli in individuals with BED, indicating the presence of motivational biases towards food. Most of these studies focused on the attentional processes and cue reactivity. For instance, individuals with BED attend to food stimuli more quickly [14, 15] and fixate on them for longer [16, 17] compared to their weight-matched counterparts without BED. Moreover, quicker attentional engagement with food stimuli in individuals with BED is accompanied by elevated physiological arousal [18] and followed by heightened subjective craving [19]. Similarly, greater cue reactivity, measured as greater neural activations in the reward circuitry, physiological responses, and subjective craving, has been found to predict greater unhealthy eating behavior and weight gain [20]. However, these previous studies did not directly assess reward associative learning in individuals with BED, which is one of the core mechanisms underlying the compulsiveness of binge-eating as postulated in the food addiction model.

### The Pavlovian-instrumental transfer (PIT) paradigm

The PIT paradigm is a widely used experimental paradigm to model the influence of conditioned cues on instrumental responses and can be used to study individual differences in motivational biases following cue exposure [21]. The PIT paradigm consists of a training phase in which the participants learn the association between a stimulus and an outcome (S–O) via classical conditioning and the association between a response and an outcome (R–O) via instrumental conditioning. In the PIT testing phase, participants are asked to respond, typically using key presses, to obtain rewards based on the learned instrumental associations, in the presence or absence of conditioned cues. The PIT transfer effects refer to the extent to which the presence of conditioned cues biases instrumental responding. A specific transfer effect refers to the biased instrumental responding for the signaled reward, i.e., an increase in specific approach motivation. A general transfer effect refers to the biased instrumental responding towards any reward, i.e., an increase in general approach motivation. The PIT paradigm with food rewards in conjunction with satiation procedures can be used to examine whether the cue sensitization effect persists after outcome devaluation.

Two previous studies have used the PIT paradigm in conjunction with food stimuli to study the influence of cue exposure on food-seeking behavior in individuals with obesity; however, they did not find significant differences in the PIT transfer effects in those with obesity compared to their normal-weight counterparts [22, 23]. On the other hand, Lehner et al. [24] found significantly stronger transfer effects in individuals who were overweight relative to those who were normal-weight or those with obesity. These mixed findings might be attributable to the limitation that binge-eating was not measured and controlled. As argued by Meule [25], addiction processes may not apply to the majority of individuals with obesity but only to those with binge-eating. However, there have not been any studies on the PIT transfer effects in individuals with recurrent binge-eating.

### The present study

Taken together, reward associative conditioning processes underlying addiction have been postulated to explain the initiation and perpetuation of binge-eating. However, these processes have not been empirically evaluated in individuals with recurrent binge-eating. Hence, the present study aimed to compare these processes, measured using the PIT paradigm, in individuals with recurrent binge-eating and those without. Based on the food addiction model of binge-eating, it was hypothesized that hyperpalatable food would trigger specific transfer effects such that conditioned cues would bias instrumental responses for the signaled food even after satiation, and this effect would be stronger in individuals with recurrent binge-eating.

## Method

### Participants

As this is the first study to evaluate the difference in PIT transfer effects between individuals with binge-eating and those without, we did not have an estimate for the expected effect size. Therefore, we aimed for a sample size that could detect a small-to-medium or larger effect based on relevant parameters derived from a previous study using the PIT paradigm on a healthy sample [26]. A priori power analysis indicated that a minimum sample size of 86 individuals would be required to have 90% power for detecting a small-medium effect size (repeated-measures within-between interaction effect, partial eta-square = 0.02, correlation between repeated measures = 0.60). The eventual sample consisted of 101 participants (mean age: 23.95 [SD = 5.62], % female = 76.2%).

The inclusion and exclusion criteria for all participants were: (1) aged 18 to 45 years, (2) not having any self-reported current or history of mental disorders to restrict the potential impact of other mental disorders on PIT effects (exclusive of depression and anxiety disorders as they are highly comorbid of disordered eating), (3) not having current moderate or severe levels of mood symptoms as assessed by the Depression, Anxiety, and Stress Scales (DASS-21; [27] to restrict the potential impact of current mood episodes on PIT effects, (4) not currently taking any psychiatric medications, (5) not having allergies or aversion to the experimental food stimuli, and (6) absence of active suicidality assessed by a follow-up clinical interview upon the indication of a score > 1 on item 9 of the Patient Health Questionnaire-9 (PHQ-9; [28]). Half of the sample belonged to the binge-eating (BE) group and the other half to the healthy control (HC) group. In addition to these inclusion and exclusion criteria, the binge-eating (BE) group participants had to have either a diagnosis of BED or BN as determined by the Eating Disorder Assessment for DSM-5 (EDA-5; [29]. The HC group had no current eating disorder diagnosis or symptomatology as indicated by the composite score on the Eating Disorders Symptoms Scale (EDDS) < 16.5 [30] and no self-reported lifetime eating disorders.

All participants were compensated approximately 8 USD for participating in the study. Informed consent was obtained prior to participation. Participants were informed that this study investigated the relationship between food cue reactivity and disordered eating. Institutional ethics approval was obtained prior to data collection.

### Procedures

Participants were recruited using the convenience sampling method. The study was advertised via mass email sent to all staff, students, and alums of the university. Interested individuals completed an electronic informed consent form and an initial screening survey including questions on demographics, self-report diagnosis of mental health disorders and medication use, food allergies, liking of the food to be used in the experience, the DASS-21, the PHQ-9, and the EDDS to evaluate initial eligibility. Potentially eligible participants for the BE group were followed up with the EDA-5. Those who indicated any suicidal thoughts on the PHQ-9 were followed up with an additional suicidal assessment. All eligible participants were invited to the laboratory to partake in an hour-long study involving the PIT paradigm and complete measures of covariates, including the Barrett Impulsivity Scale (BIS; [31], the computerized go/no-go task [32], and the 3-back working memory task [33]. They were all instructed to not consume any food or engage in intense physical activity within two hours prior to the experiment.

### Measures

#### EDA-5

The electronic version of the EDA-5 was used to diagnose BED and BN. The EDA-5 assesses the diagnostic criteria outlined in the 5^th^ edition of the Diagnostic and Statistical Manual of Mental Disorders [2]. The EDA-5 has been validated against the widely used, validated Eating Disorder Examination [34] and has shown to have good test–retest reliability [29]. The electronic version of the EDA-5 employs an algorithm to select subsequent questions based on answers to previous questions. When administered by bachelor-level trainees, it has shown good convergence with the EDA-5 administered in a clinical interview with *kappa*s above 0.94 and 0.82 for BN and BED, respectively [29]. In the present study, the EDA-5 was administered by author LTT, trained and supervised by author WSC, a licensed clinical psychologist. As the EDA-5 was not available in Cantonese, the native language of the current sample, the EDA-5 was translated into Cantonese by the authors, and back-translated to English by another bilingual research intern. The team then discussed any discrepancy between the back-translated version and the original version and arrived at a Cantonese version that retained the meaning closest to the original version of EDA-5. Participants diagnosed by the EDA-5 to have BED or BN were included in the BE group. A significant proportion of interviewed participants met all DSM-5 outlined diagnostic criteria for BED and BN except the criterion of having objective binge episodes (OBE, they reported having subjective binge episodes (SBE instead. We decided to include these individuals in the sample because SBE is considered a clinically useful feature in diagnosing BED and BN [35] and is also a diagnostic feature in the International Classification of Disorders-11 (ICD-11) [36].

#### EDDS

The EDDS-DSM-5 was used to assess eating disorder symptoms [30]. The questionnaire consisted of 23 items assessing cognitive and behavioral symptoms of BED, BN, and anorexia nervosa (AN) based on the DSM-5 diagnostic symptoms and criteria. The composite score computed from items 1–18 was used to screen out individuals with significant eating disorder symptoms. The EDDS has been validated in a sample of Hong Kong Chinese adolescents and had acceptable and good reliability and validities [37]. A Chinese version of the EDDS was used [26]. The internal consistency of this version of EDDS was acceptable with Cronbach’s *α* equaled 0.76.

#### BIS

The BIS is a well-validated self-report questionnaire on trait impulsivity [31]. It consists of 30 items on a four-point Likert scale where higher scores indicate higher levels of impulsivity. It measures three dimensions of impulsivity, namely, attentional, motor, and non-planning impulsivity. A validated Chinese version of the BIS was used in the present study [38]. The BIS full scale and subscales’ Cronbach’s *α*s were between 0.57 and 0.79.

#### DASS-21

The validated Chinese version of the DASS-21 was used [39]. The DASS-21 consists of 21 items assessing symptoms of depression, anxiety, and stress on a four-point Likert scale. The subscales of depression (score > 6) and anxiety (score > 5) were used to screen out elevated levels of depression and anxiety. The DASS-21 depression and anxiety subscales’ Cronbach’s *α*s were between 0.81 and 0.90, respectively.

#### PHQ-9

A validated Chinese version of the PHQ-9 [40] was used to assess participants’ depressive symptoms and suicidality. Individuals who scored a 1 or above on the question on suicidal thoughts (“Thoughts that I would be better off dead, or of hurting myself”) were followed up by a clinical interview conducted by the author WSC. The PHQ-9’s Cronbach’s *α* was 0.87.

#### Liking of food used in the PIT paradigm

Four food items were used in the PIT paradigm. Two were considered hyperpalatable, namely m&m’s chocolates and gummy candies. The other two were considered non-hyperpalatable, namely saltine crackers and raw almonds. Participants were asked their liking of each of the foods on a 4-point scale (*1—I dislike it and will not eat it; 2—I dislike it but I will eat it when offered; 3—I like it; 4—I like it a lot*). Participants who rated any of the foods 1 were excluded from the experiment. Two sum scores of the liking of the hyperpalatable food and the non-hyperpalatable food were computed.

#### The PIT paradigm

A food-stimuli PIT paradigm was used. The PIT paradigm was programmed using the PsychoPy v3.2 software package and administered to each participant individually on a desktop computer with a 24-inch monitor in a quiet room. Participants were told that they would be presented with food pictures and actual food rewards and asked to follow the on-screen instructions to obtain food rewards. Each participant was asked to use their dominant hand to press the keys and pick up the food items throughout the PIT. The food items were placed to the side of the computer monitor corresponding to the participant’s dominant hand.

**Fig. 1:**
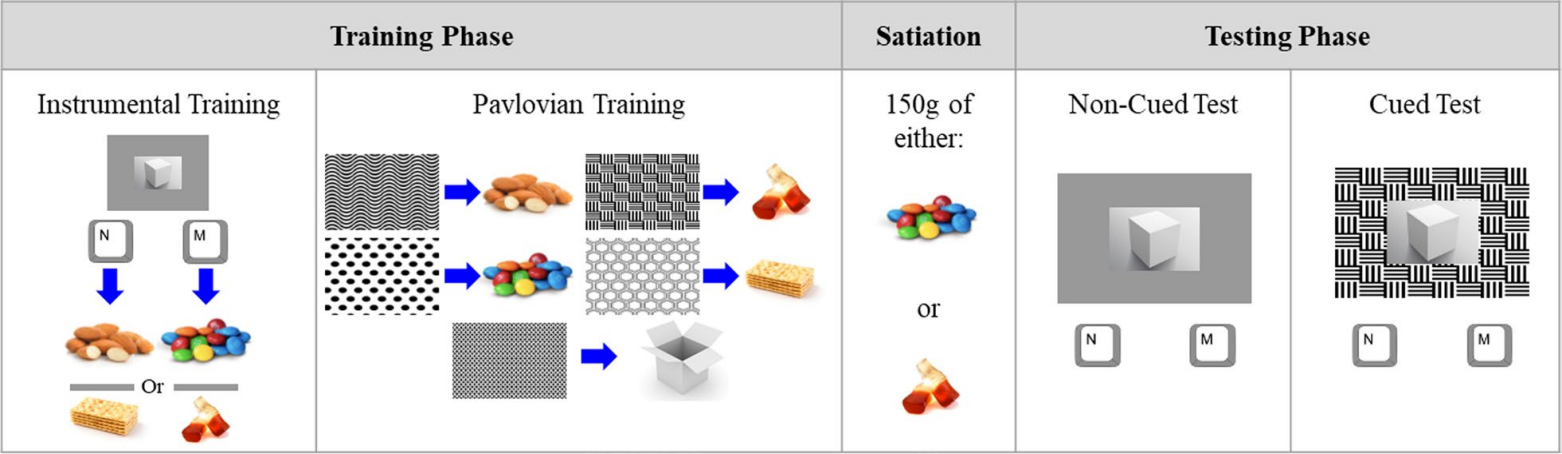
Pavlovian-Instrumental Transfer (PIT) Paradigm. Each participant learned the response-outcome associations between two key presses and two food outcomes, one hyperpalatable (O1), one non-hyperpalatable (O2), and the stimulus-outcome associations between five neutral graphical cues and five outcomes. Two cues were associated with the two food outcomes (O1, O2) trained to be linked to the two keys, i.e., specific food-predicting cues (S1, S2). Two cues were associated with the two food outcomes (O3, O4) not seen in instrumental training, i.e., general food-predicting cues (S3, S4). One cue was associated with a no-food outcome (O5) serving as the no-reward cue (S5). Non-cued and cued tests were conducted to evaluate response biases and response vigor in the presence of no cue, specific cues, general cues, and no-reward cues, following satiation on the hyperpalatable food consumed in instrumental training

As shown in Fig. 1, the PIT paradigm consists of an instrumental training phase, a Pavlovian training phase, satiation, and a testing phase. During the instrumental phase, the participants learned the R-O association between pressing “m” and “n” keys, i.e., response 1 (R1) and response 2 (R2) to one hyperpalatable food (O1) and one non-hyperpalatable food (O2) through trial and error. They were instructed to repeatedly press either “m” or “n” on a keyboard with the index finger of their dominant hand in each trial to reveal one of the two food items stored inside the white box, represented on the computer screen. They were told that the faster they press either of the keys, the more likely they would reveal the food item inside the box. A variable ratio schedule of 10 was used, i.e., after 5 to 15 presses of a key, an image of the associated food reward appeared on the screen for 1 s. Every fourth display of a food outcome on screen would be accompanied by on-screen instructions and a “ding” sound, prompting the participant to taste the corresponding bite-sized food item. The association between the keys and the food outcomes was counterbalanced. There were four blocks containing 24 trials in total and four query trials that were administered towards the end of the second and fourth blocks, which tested the participants’ knowledge of the R–O associations.

In the Pavlovian training phase, the participants learned the associations between five black-and-white neutral graphical patterns and five outcomes, i.e., the S–O associations. Two were specific food-predicting cues (S1, S2); they were associated with the hyperpalatable food (O1) and the non-hyperpalatable food (O2) used in the instrumental training phase, respectively. Two were general food-predicting cues (S3, S4); they were associated with the hyperpalatable food (O3) and non-hyperpalatable food (O4) not used in the instrumental training phase, respectively. One cue was the no-reward cue (S5); it was associated with a no-food “white box” outcome (O5). The participant was asked to attend to the screen but was not required to press any keys. The graphical cue appeared on the screen for 2 s and was then overlaid with the picture of the associated outcome for 1 s. Every fourth time that a food outcome was presented, the participant was prompted by a “ding” sound and on-screen instruction to consume the food. There were five blocks containing a total of 50 trials. Each of the five Pavlovian cues was displayed twice within each block. Query trials were used to test the participant’s knowledge of the S–O associations at the end of the third and fifth blocks.

Following the training phases, the participant undertook the outcome devaluation procedure, i.e., satiation. They were given a bowl containing 150 g of the hyperpalatable food they were exposed to during instrumental training. The participant had 10 min to consume the food and was told to eat until they felt full. A 10-min section from a nature documentary was played during the satiation phase to simulate a natural and relaxing environment where individuals would eat while watching television.

Following satiation, the PIT testing phase was conducted. The participant was asked to press either the m or n key to obtain the food reward they wanted. They were told that although no food rewards would be shown on screen or rewarded immediately, they would get the rewards at the end of the experiment. Two demo non-cued trials were administered to all participants preceding the non-cued and cued tests. The non-cued test was composed of 10 trials in total. During the non-cued test, a white box was presented for 3 s in each trial against a blank grey background during which the participant had to press either m or n to earn the food outcome. The cued test was composed of 40 trials in total (i.e., four blocks of ten trials). In the cued test, the participant was asked to press either the m or n key in each trial to earn the food rewards, yet whilst they were doing so, one of the five Pavlovian cues (S1–S5) would be displayed behind the white box (all presented for 3 s). They were told not to pay attention to the background patterns and focus on pressing either key to earn their food reward. Each block contained two presentations of each Pavlovian cue. The numbers of presses of m and n during the two tests were recorded.

#### Hunger

Participants were asked to rate their hunger on a visual analog scale prior to the PIT paradigm. They were presented with a horizontal line with a 0 on the left end and 100 on the right end on the computer screen, and asked to move the anchor along the line to indicate their hunger levels.

#### Go/no-go task

Following the PIT paradigm, the participant completed the computerized go/no-go task [32] to assess response disinhibition. The go/no-go task consisted of four blocks of go/no-go trials totaling 100 trials. Each block consisted of 20 “go” trials and five “no-go” trials. The participants were asked to press a key to indicate the number they saw in each trial was a go signal and not press a key if it was a no-go signal. They received feedback after each trial stating “correct” or “incorrect”. The number and the feedback stayed on the screen for 1 s, each. The inter-trial interval was 1 s. The percentage of hit and correct rejection trials was used as a covariate.

#### N-back working memory task

The participants also completed a 3-back working memory task to assess working memory [33]. The 3-back working memory task consisted of two blocks of 20 trials each, totaling 40 trials. The participants had to determine if the letter presented in a trial matched with the letter presented three trials prior. In each trial, a number appeared and the participants had 3 s to respond. If the response was correct, the number turned green. If the response was incorrect, the number turned red. The inter-trial interval was 1 s. The percentage of correct trials was used as a covariate.

### Statistical analysis

#### Overview

All the analysis was conducted in SPSS version 28.0. Descriptive statistics were examined to see if any demographic and psychological variables differed between the BE and HC groups. Mixed ANOVAs were conducted to determine if specific food-predicting cues biased instrumental responses for the signaled food, i.e., specific transfer effects, if general food-predicting cues elevated response vigor in general, i.e., general transfer effects, and if these effects differed by group and food palatability. Tests of normality, sphericity, and homogeneity were conducted to evaluate if the data met the assumptions required for mixed ANOVAs and if adjustments were needed. Evaluations of the residual plots suggested that the normality assumption was met. The Mauchly’s test of Sphericity was significant, indicating that the sphericity assumption was violated; hence, the Greenhouse–Geisser correction was applied to the degrees of freedom for the within-subjects tests. The Levene’s tests of equality of error variances were non-significant, suggesting that the homogeneity assumption was met.

#### Specific transfer effect

Similar to the approach taken by Watson et al. [23], a 2-way (cue × group) mixed ANOVA was conducted to determine if the specific food-predicting cue biased the instrumental responses for the signaled hyperpalatable food compared to the no-reward cue, and whether this effect differed by group. The percentage of instrumental responses for the signaled hyperpalatable food was the dependent variable. Given that an increased percentage of instrumental responses could be caused by decreased responses to non-signaled food, increased responses to the signaled food, and/or decreased responses to the no-reward cue [41], we also examined the rate of responding (the average number of key presses per trial) in the non-cued test compared to the rate of responding in the presence of specific food-predicting cue and no-reward cue. Specifically, a 3-way (cue × group × food type) mixed ANOVA was conducted to evaluate the effect of cue and its interaction with group and food type. The specific transfer effect was examined by comparing the rates of responding in four scenarios: (1) baseline rate of responding for the to-be-signaled food in the non-cued test, (2) the rate of responding for the signaled food in the presence of the outcome-specific cue, (3) the rate of responding for the non-signaled food in the presence of outcome-specific cue, and (4) the rate of responding for the signaled food in the presence of the no-reward cue. Within-individual contrasts were conducted for the within-individual effect of cue such that the baseline response was the reference point. Food types referred to the devalued hyperpalatable and the still-valued non-hyperpalatable food.

#### General transfer effect

Although we did not have a hypothesis regarding general transfer effects, we explored whether there would be any group differences in general transfer. A 2-way (cue × group) mixed ANOVA was conducted to evaluate the general transfer effect and its interaction with group. The general transfer effect was determined as the increased response vigor, computed by summing the number of both key presses by trial in the presence of general food-predicting cues in comparison to the response vigor at baseline in the non-cued test and in the presence of the no-reward control cue.

## Results

### Descriptive statistics

Of the 101 participants, 51 were in the BE group and 50 the HC group. Within the BE group, 15 were diagnosed with BN, 20 with BED, two meeting all criteria of BN and 14 meeting all criteria of BED but had only SBE and not OBE. Participants’ demographic information, baseline psychological measures scores, and cognitive task performance scores are presented in Table 1. As shown in Table 1, the two groups did not differ in age and BMI. The HC group consisted of a greater proportion of women. As expected, the BE group had greater depressive and anxiety symptoms and eating disorder symptoms. The BE group also had greater BIS attentional impulsivity. The two groups did not differ significantly in subjective liking of hyperpalatable and non-hyperpalatable food, hunger before the PIT testing phase, go/no-go task, or the 3-back working memory task. Regardless of group, hyperpalatable food was liked more strongly than non-hyperpalatable food (main effect of palatability: *F*[1,99] = 24.51, *p *< 0.001, group by palatability interaction: *F*[1,99] = 1.01, *p* = 0.32). The BE subgroup with OBE and the BE subgroup with SBE did not differ on these variables except that the OBE group had a significantly greater EDDS composite score than the SBE group (*t* = 2.18, *p* = 0.034). Hence, the subsequent analyses were conducted in both the full sample and the subsample with HC and OBE only. The results from these two sets of analysis were largely the same. Therefore, the results of the analysis with the full sample was hereby reported.

**Table 1:** The demographic characteristics and psychological measures of the participants in their respective groups

	BE	HC	*t*-test
	(n = 51) (*M*/*SD*)	(n = 50) (*M*/*SD*)	*t*(99) (*t*/*p)*
Age	23.37 (5.50)	24.54 (5.72)	1.045 (0.30)
% of females	62.00%	90.20%	11.08 (0.001)^a^
BMI	22.24 (3.79)	21.89 (4.31)	− .36 (0.67)
PHQ total score	10.98 (4.88)	5.06 (3.58)	− 6.96 (< 0.001)
DASS total score	23.92 (11.71)	9.16 (7.58)	− 7.48 (< 0.001)
DASS anxiety score	6.30 (4.01)	2.34 (2.35)	− 6.03 (< 0.001)
DASS depression score	7.70 (4.91)	2.56 (2.72)	− 6.47 (< 0.001)
DASS stress score	9.92 (4.69)	4.26 (3.36)	− 6.94 (< 0.001)
EDDS composite score	47.75 (14.47)	8.58 (5.42)	− 18.08 (< 0.001)
BIS total score	68.00 (10.23)	65.36 (7.98)	− 1.44 (0.15)
BIS attentional score	18.69 (4.25)	17.14 (3.34)	− 2.03 (0.045)
BIS motor score	22.61 (4.07)	22.12 (3.52)	− .64 (0.52)
BIS non-planning score	25.78 (4.86)	24.98 (3.73)	− .93 (0.35)
Liking of hyperpalatable food	6.12 (0.93)	6.26 (0.80)	.82 (0.41)
Liking of non-hyperpalatable food	5.63 (0.96)	5.52 (0.93)	− .57 (0.60)
Hunger prior to PIT testing	35.47 (28.29)	29.40 (23.12)	− 1.18 (0.24)
Go no go task (% correct)	97.0 (2.3)	96.6 (2.2)	− 0.81 (0.42)
3-Back working memory task (% correct)	78.7 (9.8)	81.7 (15.2)	1.21 (0.23)

*BE* Binge-eating group, *HC* Healthy control group

^a^Chi-square test

### Learning outcomes in training phases

Participants in the BE group achieved 94% correctness in the R-O knowledge query trials whereas compared to 97% of the HC group. The difference was not statistically significant (*t* = 1.17, *p* = 0.25). Similarly, the BE group achieved 97% correctness in the S–O knowledge query trials compared to 99% and the difference was not statistically significant (*t* = 1.84, *p* = 0.07).

### Outcome devaluation

As expected, when participants were sated on hyperpalatable food, they pressed more of the key associated with the non-hyperpalatable food (8.29 presses per trial) than the key with the hyperpalatable food (5.85 presses per trial) in the non-cued test (paired-sample *t* = 2.81, *p* < 0.01). On average, 59% of the key presses were for the non-hyperpalatable food in the non-cued test.

### Specific transfer effect

**Fig. 2:**
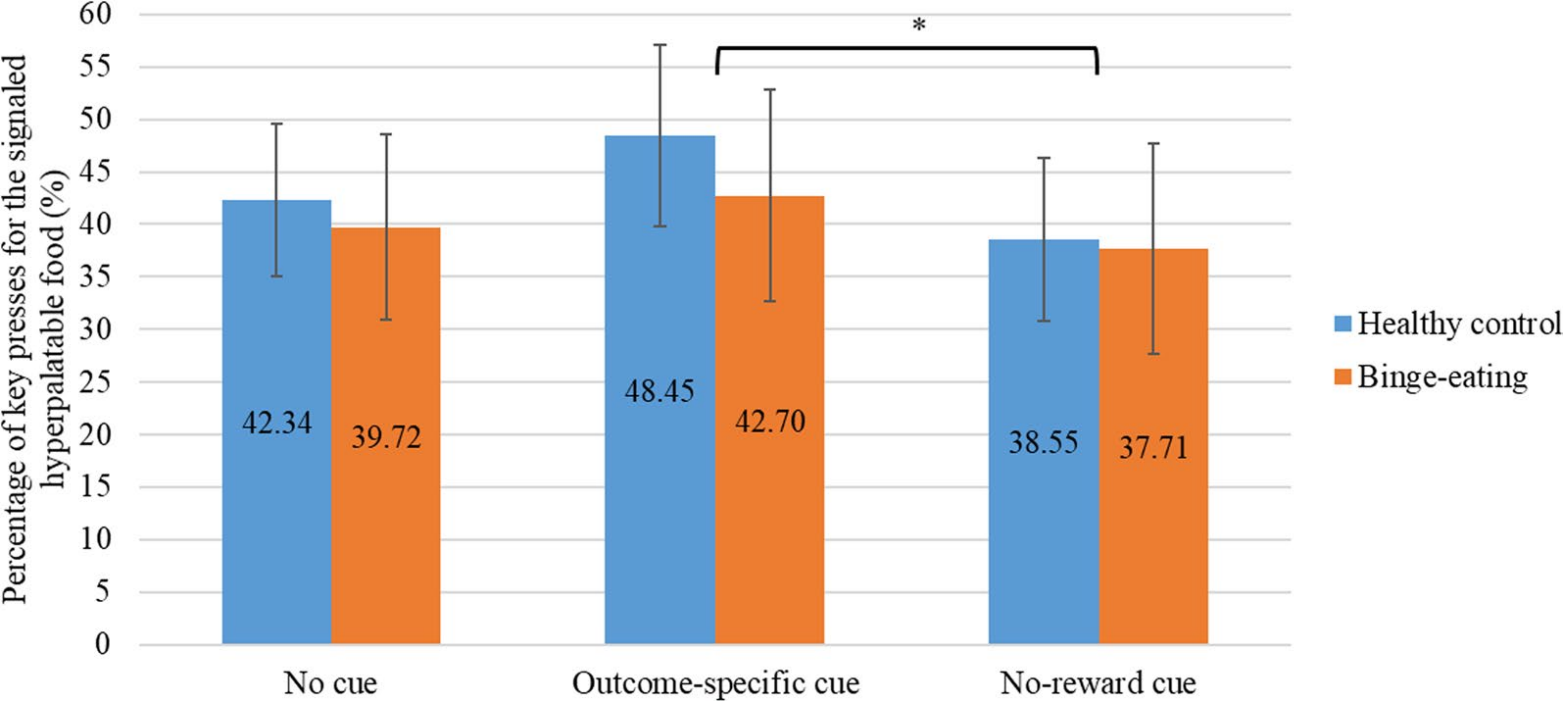
Biased Instrumental Responding for Signaled Hyperpalatable food. This figure illustrates the percentages of key presses for the signaled hyperpalatable food by cue type and group. There is no significant cue type by group interactions but a significant main effect of cue type. Error bars indicate 95% confidence intervals. Values inside bars are mean vales. *indicates a significant difference between the percentages of key presses for the signaled hyperpalatable food in the presence of the outcome-specific cue compared to the cue predicting no reward

The 2-way (cue × group) mixed ANOVA with the percentage of instrumental responses for the signaled hyperpalatable food as the dependent variable showed a non-significant cue × group interaction effect (*F*[2,98] = 0.42, *p* = 0.66), suggesting that the biasing effect of Pavlovian cues on instrumental responses did not differ by group. The main effect of cue was significant (*F*[2,98] = 3.82, *p* = 0.03). As expected, the percentage of instrumental responses for the signaled hyperpalatable food in the presence of the specific food-predicting cue was significantly higher than that in the presence of the no-reward cue (*F*[1,99] = 7.61, *p* < 0.01); see Fig. 2).

**Fig. 3:**
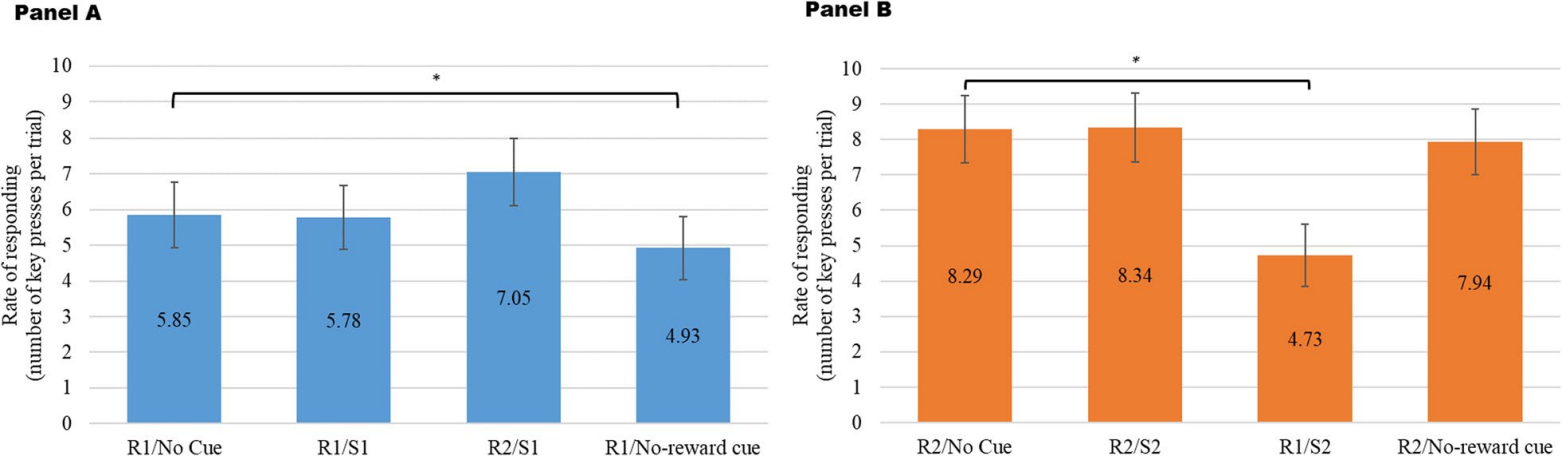
Rates of Instrumental Responding for Signaled and Non-Signaled Food by Cue Type. This figure illustrates the number of key presses per trial in the absence of cues and in the presence of different Pavlovian cues. R1—key press for the devalued hyperpalatable food; R2—key press for the still-valued non-hyperpalatable food. S1—outcome-specific cue predicting the devalued hyperpalatable food. S2—outcome-specific cue predicting the still-valued non-hyperpalatable food. Error bars indicate 95% confidence intervals. *p < .05

To further evaluate if the biased responding was due to increased responses for the signaled outcome, suppressed responses for the non-signaled outcome, or suppressed responses in the presence of the no-reward cue, a 3-way (cue × group × food type) mixed ANOVA was conducted with the rate of responding as the dependent variable. The 3-way interaction was non-significant (*F*[3,97] = 0.04, *p* = 0.99). However, the cue × food type 2-way interaction was significant (*F*[3,97] = 3.77, *p* = 0.01) suggesting that the main effect of cue differed depending on which food outcome was signaled (see Fig. 3). Tests of 2-way (cue × palatability) within-individual contrasts showed that the interaction effect was attributable to the difference between the baseline rate of responding (no cue) and the rate of responding for the non-signaled food in the presence of outcome-specific cue (*F*[1,99] = 8.90, *p* = 0.004). As shown in Fig. 3, the rate of responding for the non-signaled food was significantly suppressed compared to the baseline rate when the signaled food was the still-valued non-hyperpalatable food (*F*[1,99] = 18.63, *p* < 0.001); see Panel B) but this difference was not significant when the signaled food was the devalued hyperpalatable food (see Panel A). Additionally, the rate of responding was significantly suppressed in the presence of the no-reward cue compared to baseline when the signaled food was the devalued hyperpalatable food (*F*[1,99] = 6.88, *p* = 0.01). The main effect of group was not significant (*F*[1,99] = 2.15, *p* = 0.15).

### General transfer effect

**Fig. 4:**
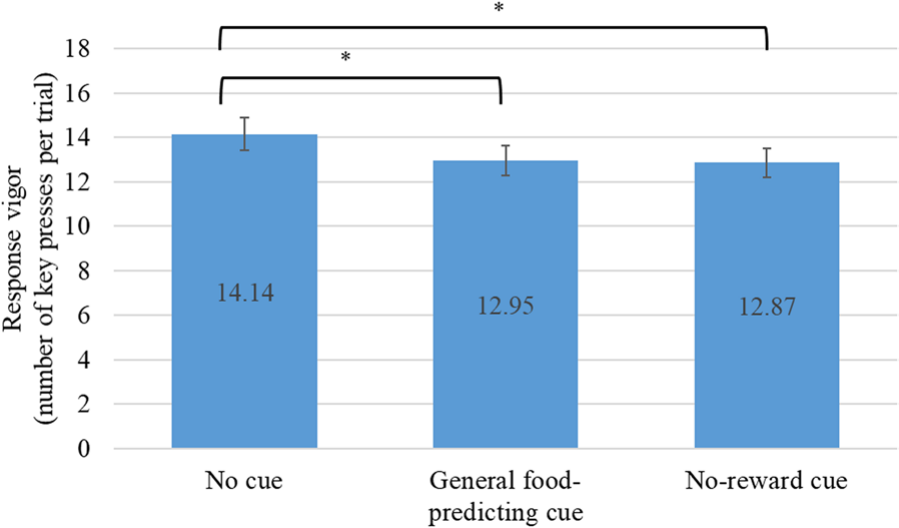
Response Vigor at Baseline and in the Presence of Pavlovian Cues. Error bars indicate 95% confidence intervals. *Indicates a significant difference

A 2-way (cue × group) repeated-measures ANOVA was conducted to determine if the response vigor in the presence of general food-predicting cues was elevated compared to baseline response vigor and the response vigor in the presence of the no-reward cue. The 2-way interaction effect was not significant (*F*[2,98] = 1.01, *p* = 0.37). The main effect of cue was significant (*F*[2,98] = 13.12, *p* < 0.001; see Fig. 4). The main effect of group was not significant (*F*[1,99] = 1.55, *p* = 0.22). Contrary to the expectation, within-subject contrasts with the baseline response vigor as the reference point showed that the response vigor was significantly suppressed in the presence of general food-predicting cues (*F*[1,99] = 26.19, *p* < 0.001) and the no-reward cue (*F*[1,99] = 24.53, *p* < 0.001).

## Discussions

### General findings

The present study examined Pavlovian-instrumental transfer effects in individuals with recurrent binge-eating compared to healthy controls. Contrary to the hypothesis, we did not find significant differences in transfer effects by group. We did find significant main effects of cue, contributed by the suppressed rate of responding either in the presence of the no-reward cue or the suppressed rate of responding to the non-signaled food. Furthermore, the presence of general food-predicting cues did not increase response vigor. Contrary to our expectations, response vigor decreased in the presence of Pavlovian cues.

### Transfer effects

By examining the percentage of instrumental responses for the signaled food, we found specific transfer effects represented in greater biased responding for the signaled food in the presence of the specific food-predicting cue compared to that in the presence of the no-reward cue (see Fig. 2). Further examination of the rates of responding revealed that the biasing effect of Pavlovian cues was not due to increased responding in the presence of outcome-specific cues, but to the suppressed responding in the presence of the no-reward cue and the suppressed responding for the non-signaled food (see Fig. 3). This pattern of differential rates of responding appeared to contradict the elevated responding for the signaled food observed in previous studies [23, 42, 43]. We believe that these inconsistent results may be attributable to variabilities in experimental procedures and analytical approaches. For instance, we did find the biasing effect of the Pavlovian cues similar to that in Watson et al. [42], it was only when we examined the rates of responding across cue types did we find that the biasing effect was due to suppressed responding. The procedures and analytical approach in Watson et al. [23] were different in that priming rates were examined rather than rates of responding. We used the same analytical approach as in Quail et al. [43], however, the food outcome was not devalued in their study. As specific transfer effects could be attenuated by outcome devaluation [41], the satiation procedure in the present study might have led to suppressed responses for the devalued hyperpalatable food, especially in the presence of the cue predicting the still-valued non-hyperpalatable food. Moreover, the order of the non-cued and cued testing trials in Watson et al. [42] was counterbalanced, with a break in between. In the present study, the non-cued trials were presented first, followed by the cued trials without a break. As the transfer tests were conducted in extinction with no rewards given immediately, the first-presented set of non-cued tests could have decreased the strengths of the R-O associations and led to decreased responding in the subsequent cued trials. Without a break, there could also be a fatigue effect on responding in the cued trials. Hence, responding in the cued trials could have been suppressed due to this order effect. Future studies presenting a randomized order of non-cued and cued trials could eliminate the order effect and better evaluate food-related specific transfer.

In the examination of general transfer effects, contrary to our expectations, we found suppressed response vigor in the presence of general food-predicting cues and the no-reward cue compared to the baseline response vigor. In addition to the abovementioned order effect, we speculate that the suppressed responding might be attributable to increased mental processing of the S–O associations during the cued test compared to the non-cued test. Although we explicitly asked the participants not to pay attention to the cues, it is still highly plausible that they did and the increased mental processing might have led to greater latencies in responding, and hence fewer key presses in total.

### PIT effects in HC versus BE groups

We did not find any main effect or interaction effect with group in the present study. This finding contradicted the primary hypothesis that individuals with recurrent binge-eating would have greater vulnerability to sensitization by food cues and would engage in greater food-seeking behavior following conditioned cue exposure. We propose several mechanisms that may explain this null finding. First, the BE group in the present sample consisted of some individuals who had SBE but not OBE. Previous findings on the distinction between individuals with SBE and OBE were inconclusive; they were found to have similar levels of negative affect and disordered eating symptoms but different personality characteristics [44], differential responses to treatments [45], and consumption of different proportions of hyperpalatable food during binge episodes [46]. In our sample, the OBE subgroup had significantly greater EDDS composite scores, however, we conducted all analyses with the subsample consisting of HC and BE with OBE and found the same results. Nonetheless, the potential differences in reward conditioning processes between individuals with OBE and SBE remain to be empirically evaluated in future studies with greater statistical power.

Second, the PIT effects might be modulated by cognitive and inhibitory control [47]. The dual process models of binge-eating posited that binge-eating is the result of two interacting systems: the impulsive system and the reflective system [48, 49]. While greater vulnerability to cue sensitization might predispose a person to binge-eating, the reflective system may be capable of offsetting this effect. Binge-eating may only occur when heightened cue sensitization is coupled with impaired inhibitory control. In the present sample, only minimal differences were observed in BIS attentional impulsivity but not in other domains of impulsivity nor the overall BIS scores. The performance in the go/no-go task, a measure of response disinhibition, was also not significantly different between groups. It is possible that the BE group in the present study consisted of high-functioning community-dwelling individuals whose inhibitory control was intact; their vulnerability to sensitization by food cues might exhibit only under circumstances when their inhibitory control is impaired.

Third, the vulnerability to cue sensitization in individuals with binge-eating may be triggered only when they are exposed to idiosyncratic food or contextual cues that have been associated with previous episodes of binge-eating. Previous research on a food cue exposure intervention for individuals with obesity found that participants reduced eating in response to the exposed food but not in response to other food [50], suggesting that motivational biases elicited by conditioned cues may be restricted to specific cues associated with one’s own previous eating experience, which could not be simulated in a brief experimental paradigm such as the PIT. Future studies using idiosyncratic food items, such as self-selected “binge food,” might enhance the ecological validity of the PIT paradigm and might be more able to detect individual differences.

### Food addiction model

The hypothesis of the present study was formulated based on the food addiction model of binge-eating which postulates that binge-eating is perpetuated by similar mechanisms underlying drug addiction, such as cue sensitization and the transition from goal-directed behavior to habitual actions [6, 7]. The present finding did not support this hypothesis. It should be noted that, within the field of substance addiction, whether people with drug abuse have impaired goal-directed behavior leading to greater habitual actions remains controversial, along with mixed results regarding specific transfer effects in people with and without drug abuse [51]. In particular, Hogarth et al. [51] proposed that addiction might be driven by excessive goal-directed actions to cope with adverse motivational states, e.g., negative mood, depression, and withdrawal, rather than excessive habit learning, and that individuals with drug addiction may have a sensitivity to adverse motivational states. Indeed, sensitivity to loss is found to be heightened in individuals with BN [52]. Future research may evaluate not only appetitive PIT effects but also inhibitory PIT effects in response to negative outcomes.

### Limitations

Our findings should be interpreted with consideration of the following limitations. First, all participants were recruited primarily from the university population; our findings might have limited generalizability to the general population with more diverse demographic and educational backgrounds. Second, although we assessed binge-eating with the validated EDA, all participants in the BE group were recruited from the community, and most of them were university students. They may not represent clinical populations with binge-eating. Additionally, the EDA version we used was translated to Cantonese by this research team. This Cantonese version had not been validated against clinical interviews in the local population. Future studies employing a locally validated Cantonese version of the EDA-5 would improve the methodological rigor. Third, we included participants who had SBE but not OBE, which introduced greater heterogeneity in the sample and could have decreased the power to detect group differences. Fourth, the experiment was not conducted at a specific time within a day. The timing of the day can influence food preferences and appetite [53]. Circadian effects could have confounded our results. Finally, the hormonal cycle of female participants can influence food craving and food intake but was not controlled in the present study. Future studies should control the timing of the menstrual cycle when the experiment is conducted.

### Conclusions and future research directions

This is the first study that examined the Pavlovian-instrumental transfer effects in individuals with recurrent binge-eating. The present findings did not support the hypothesis that specific transfer effects were stronger in individuals with binge-eating, and the observed transfer effects were explained mostly by suppressed instrumental responding in the presence of Pavlovian cues predicting no reward. Future studies employing more ecologically valid designs of the Pavlovian-instrumental transfer paradigm and investigating inhibitory PIT effects may enhance the understanding of motivational processes underlying binge-eating.

## Data Availability

All de-identified data and codes are available upon request for research and review purposes.
